# Rapid *in situ* formation of a double cross-linked network hydrogels for wound healing promotion

**DOI:** 10.3389/fphar.2025.1562264

**Published:** 2025-03-18

**Authors:** Yifan Liu, Ye Zhang, Qianqian Jia, Xiaoyun Liang, Kejin Xu

**Affiliations:** ^1^ College of pharmacy, Changchun University of Chinese Medicine, Changchun, Jilin, China; ^2^ Key Laboratory of Colloid and Interface Chemistry of State Education Ministry, Shandong University, Jinan, Shandong, China

**Keywords:** hydrogel dressing, wound healing, angelica sinensis polysaccharide, wound management, material sciences

## Abstract

The persistent challenge lies in accelerating wound healing. Bioactive hydrogels with *in situ* formation properties ensure that the dressing completely adheres to the wound and isolates it from external bacteria and microorganisms in order to meet the needs of damaged skin tissue for rapid hemostasis and wound healing. In this paper, hydrogel dressing that Polyacrylamide/Sodium alginate grafted with dopamine/Gelatin grafted with glycidyl methacrylate doped with Angelica sinensis polysaccharide was prepared (PDGA). Chemical cross-linking of PAAM by adding cross-linking agent to initiate free radical polymerization and photocross-linking by free radical polymerization of GMA-GEL under UV light irradiation are two cross-linking modes to construct dual-cross-linking network of PDGA hydrogel dressing. The hydrogel remains fluid when placed in a sealed syringe and solidify rapidly by photocross-linking when placed on the wound. Furthermore, the hydrogel demonstrated excellent biocompatibility and hematological safety. The interaction between angelica polysaccharides and integrins on the platelet surface facilitated an augmentation in platelet adhesion, activation, and aggregation, ultimately inducing rapid coagulation of the blood within 130 s in a mouse tail vein hemorrhage model. ASP can promote tissue healing by promoting cell proliferation around wounds and accelerating the formation of new blood vessels. In a mouse skin defect model, collagen deposition, blood vessel formation, hair follicle regeneration, and granulation tissue formation were observed due to the presence of angelica polysaccharides, showing significantly superior wound healing properties when compared to Tegaderm™ film. In addition, the expression of CD31 in skin wounds treated with PDGA was significantly upregulated. Consequently, PDGA multifunctional dressings exhibit considerable potential for *in vitro* hemostasis and skin wound repair applications.

## 1 Introduction

As the main barrier between the human body and the external environment, the skin plays a crucial protective role. However, it is also vulnerable to various external factors. These factors may include physical friction, high or low temperature exposure, chemical and corrosive material exposure, and biological microbial infections. These damages can often lead to different types of skin lesions, such as cuts, abrasions, burns, etc. These injuries can not only bring pain and discomfort to the patient, but also may affect their daily life and work ability, and in severe cases, they may even cause infections and other complications (Tottoli et al., 2020; Tavakoli and Klar, 2021; Ding et al., 2023). After the skin was damaged, the natural healing process is frequently constrained by diverse factors. Without taking timely therapeutic measures, the healing of wounds may be hindered or the process may be prolonged (Jones and Hampton, 2021; Li et al., 2022; Kowalski et al., 2024). Recently, numerous researchers have focused on exploring new materials capable of effectively enhancing wound healing, among which hydrogels have garnered significant attention owing to their ability to keep wounds moist and block external contaminants, among other multiple advantages (Zhang et al., 2023). Hydrogels are mainly derived from a variety of natural or synthetic hydrophilic polymeric materials each of which aims to optimize the specific properties of the hydrogel to meet the varying needs of wound healing (Kamoun et al., 2017; Francesko et al., 2018; Tavakoli and Klar, 2020; Ciolacu et al., 2023). Among these, the *in situ*-forming hydrogel offers distinct advantages, as it rapidly forms a tightly adherent protective layer at the wound site, effectively shielding it from external contaminants and pathogens. It can be used as a drug delivery system, by loading the drug and realizing controlled release and improve the stability of drugs (Agarwal et al., 2022; Feng et al., 2023; Feng et al., 2024; Villa et al., 2024). Current studies have shown that hydrogels formed *in situ* have weak mechanical properties, which may result in their inability to maintain structural stability for long periods of time, and the gelling time is often long, which may delay treatment in medical emergencies.

Polyacrylamide has been widely used in medical fields such as drug delivery, medical materials and surgical aids (Wang L. et al., 2022; Tirey et al., 2024). These applications mainly utilize the biocompatibility, degradability and a certain degree of stability. However, the application of polyacrylamide in wound dressings been limited. This is mainly because polyacrylamide its swelling properties do not meet the requirements of medical dressings, and the molecular structure of polyacrylamide is relatively simple and lacks the specific functional groups or chemical groups required for cell adhesion (Reinhards-Hervás et al., 2024). Researchers improve polyacrylamide defects by combining different materials. Gelatin has been shown to enhance the biocompatibility of acrylamide and promotes cell growth and differentiation capacity (Wang Y. et al., 2022; Chen et al., 2023). Sodium alginate possesses robust water-absorbing capabilities, enabling it to absorb exudate from the wound surface and maintain wound moisture. After the combination of polyacrylamide and sodium alginate, the water-absorbing and moisturizing performance of hydrogel is further improved, which helps to maintain the moist state of the wound and promote the proliferation and differentiation of cells (Hasani-Sadrabadi et al., 2020).

Angelica sinensis polysaccharide (ASP) is the active compound extracted from Angelica sinensis (Tian et al., 2024), with a content of up to 15% in the plant. Studies have demonstrated that angelica polysaccharides exhibit notable advantages in tissue healing, manifesting primarily in their bioactivities. ASP promotes the proliferation and differentiation of pluripotent hematopoietic stem cells and hematopoietic progenitor cells, leading to an increase in the number of peripheral blood cells, leukocytes, hemoglobin, and bone marrow nucleated cells, which ultimately accelerates the tissue healing process. Moreover, ASP serves as an immunomodulatory agent, capable of modulating the activity of complement receptors on the lymphocyte membrane and augmenting the immune response of lymphocytes, thereby exerting a positive effect on the immune function of the body. This contributes to reducing the risk of infection and facilitates wound healing (Wang et al., 2017; Bi et al., 2021; Brumberg et al., 2021; Nai et al., 2021). Despite the numerous benefits associated with angelica polysaccharides, their direct application is constrained by several factors, namely, stability issues, challenges in controlling release rates, and excessively high local concentrations. Utilizing hydrogel as a carrier for angelica polysaccharides can overcome the limitations associated with direct application and offer the following advantages: hydrogel can provide a stable microenvironment that shields angelica polysaccharides from external factors, thereby enhancing their stability. Furthermore, precise control over the release rate and concentration of angelica polysaccharides can be achieved by adjusting the composition and structure of the hydrogel, thereby enabling stable and predictable therapeutic outcomes. The hydrogel ensures that the concentration of angelica polysaccharides in local tissues remains moderate, neither excessively high to elicit adverse reactions nor too low to compromise the therapeutic efficacy.

**FIGURE 1:**
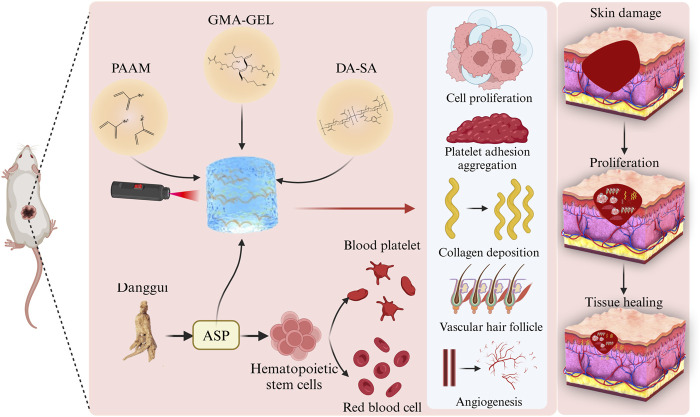
Schematic of PDGA hydrogel dressing promotion of wound healing. PDGA remodels tissues by promoting cell proliferation, platelet adhesion and aggregation, collagen deposition, angiogenesis and hair follicle generation.

The central question of this study is: can hydrogels of specific composition and properties accelerate the wound healing process by modulating the wound microenvironment? To answer this question, we propose the following hypothesis: hydrogels containing specific bioactive components can significantly improve the speed and quality of wound healing by promoting cell proliferation, platelet adhesion and aggregation as well as collagen deposition, and vascularization and hair follicle generation. In this study presents the preparation of possessing in *in situ* formation properties a hydrogel dressing (PDGA). The hydrogel’s double cross-linked network was induced through the chemical cross-linking of PAAM and the of GMA-GEL. Subsequently, the mechanical properties of the hydrogels were characterized. Furthermore, the biocompatibility of the PDGA hydrogel was assessed through blood compatibility and cytocompatibility tests. Lastly, the PDGA hydrogel was evaluated in a full-thickness skin defect model in mice for its ability to promote vascular and hair follicle regeneration, thereby facilitating wound healing. The results indicated that the PDGA hydrogel exhibited significant hemostatic and wound-healing effects on skin tissue defects (Figure 1). It allows rapid control of bleeding situations, which is particularly important in urgent hemostasis scenarios because of the urgent need for rapid, effective and safe wound treatment strategies in the operating room environment (Chou et al., 2017; Krasilnikova et al., 2022).

## 2 Materials and methods

### 2.1 Materials

Acrylamide (AAm): Purchased from Sigma-Aldrich, purity≥99%. Ammonium Persulfate (APS): Obtained from Aladin Reagent (Shanghai, China), purity≥98%. N,N,N′,N′-Tetramethylethylenediamine (TEMED): Supplied by Aladin Reagent (Shanghai, China). N,N′-Methylenebis(acrylamide) (BIS): Procured from Sigma-Aldrich, purity≥99%. 1-Ethyl-3-(3-dimethylaminopropyl)carbodiimide hydrochloride (EDC·HCl): Purchased from Aladin Reagent (Shanghai, China). N-Hydroxysuccinimide (NHS): Obtained from Aladin Reagent (Shanghai, China), purity≥98%. Dopamine hydrochloride (DA·HCl): Supplied by Sigma-Aldrich. Ammonium alginate: Purchased from Aladin Reagent (Shanghai, China). Gelatin: Obtained from BD Biosciences (Franklin Lakes, NJ, United States), type A, from bovine skin. Glycidyl methacrylate (GMA): Procured from Tokyo Chemical Industry Co, Ltd. (Tokyo, Japan), purity≥97%. Angelica sinensis polysaccharide (ASP): Supplied by Shanghai Yuanye Bio-Technology Co, Ltd. (Shanghai, China), Biological Reagent 60%. Lithium Phenyl-2,4,6-trimethylbenzoylphosphinate (LAP): Purchased from Sigma-Aldrich, purity≥98%.All solutions were prepared using ultrapure water (Milli-Q system, MilliporeSigma, Burlington, MA, United States) to ensure the highest purity and minimize contamination.

### 2.2 Methods

#### 2.2.1 Synthesis of GMA-GEL and DA-SA

GMA-GEL was first synthesized by modifying the previously reported method (Sk et al., 2021). The carboxyl and -OH groups were depleted in the condition of pH = 3.5, and the GMA molecule reacted with the carboxyl group in gelatin. The details were described in the Supplementary Material. DA-SA was synthesized by facilitating the reaction between the carboxyl group of sodium alginate and the amino group of DA. The specific process is shown in the Supplementary Material.

#### 2.2.2 Preparation of PAAM/DA-SA/GMA-GEL hydrogel

First, the acrylamide of 14 wt%, the DA-SA of 2.8 wt%, the GMA-GEL of 10 wt%, and the ASP of 5 wt% were dissolved in 10 mL deionized water under light-shielded conditions. Subsequently, 0.09 mg of MBAA and 0.36 μL of Ammonium Persulfate (APS) were added to the AM/DA-SA/GMA-GEL solution. Prior to mixing, the photo-initiator LAP was introduced into the preparatory solution to obtain the precursor solution. Next, 0.4 mL of TEMED was added to the precursor solution, which was then transferred into a glass mold. Lastly, the hydrogel pre-polymer was exposed to ultraviolet light (365 nm) for 20 s. Over a period of 3 days, the PBS solution was replaced every 6 h to eliminate unreacted monomers and initiators.

#### 2.2.3 Characterizations

The preparation of GMA-GEL and DA-SA was confirmed by nuclear magnetic resonance (^1^H NMR) and Fourier transform infrared spectroscopy (FT-IR) analyses. The swelling behavior, biodegradability and microstructure of PAAM/DA-SA/GMA-GEL hydrogels were characterized through swelling experiments, degradation experiments and scanning electron microscopy (SEM) observations. For a detailed procedure, please refer to the Supplementary Material.

#### 2.2.4 Mechanical properties of hydrogel

The modulus of these hydrogels was measured using a TA rheometer (DHR-2) and documented as a function of time (Del Giudice, 2020). Compressive stress-strain curves of PAAM/DA-SA/GMA-GEL hydrogels were obtained and analyzed (Fang et al., 2019). The ability of hydrogels to slow drug release at different pH was analyzed. For detailed procedures, please refer to the Supplementary Material.

#### 2.2.5 Hemolytic test of hydrogels

A predetermined amount of hydrogel was combined with erythrocytes and incubated for 1 hour at 37 °C. Subsequently, the absorbance at 540 nm was recorded to evaluate hemolysis (Zhao et al., 2023). For a detailed procedure, please refer to the Supplementary Material.

#### 2.2.6 *In vitro* whole blood-clotting performance

The *in vitro* whole blood-clotting test was conducted following the previously established method (Zhang et al., 2024). For a detailed protocol, please refer to the Supplementary Material.

#### 2.2.7 Hemostasis performance of hydrogels

Based on previous research, the hemostatic efficacy of the PAAM/DA-SA/GMA-GEL hydrogel was assessed using a mouse-tail amputation model (female Kunming mice, weighing 28–32 g). A method of tagging experimental animals with non-invasive ear tags was used by us (Klabukov et al., 2023). For detailed experimental procedures, please refer to the Supplementary Material.

#### 2.2.8 Cytocompatibility test of hydrogels

The cytocompatibility test was performed using the leaching solution method as described in Zangeneh et al. (2019). The detailed procedure is provided in the Supplementary Material.

#### 2.2.9 Evaluation of wound healing sites with a dorsal skin defect model in mice

To further evaluate the therapeutic effect of PDGA hydrogel on wound healing, a mouse back skin defect model was establishedused. A method of tagging experimental animals with non-invasive ear tags was used by us. Hematoxylin-eosin (H&E) staining was used to qualitatively assess the histomorphology of tissue regeneration at various time points, with a particular focus on fibroblasts and epidermal regeneration within the traumatized area. The percentage of collagen deposition was assessed by Masson staining, as described in the Supplementary Material, to gauge the extent of tissue repair. All animal experiments were conducted in accordance with the approval of the Institutional Animal Care and Use Committee of Changchun University of Traditional Chinese Medicine.

#### 2.2.10 Immunofluorescence staining

Immunofluorescence staining was performed to assess vascular regeneration during wound healing, using CD31 as a marker. The detailed procedure is provided in the Supplementary Material.

#### 2.2.11 Statistical analysis

The results were presented as mean values of replicate experiments, and statistical analysis was performed using GraphPad Prism and Origin. Data represent the mean ± SD of at least three replicates. *P < 0.05, **P < 0.01, ***P < 0.001, and ****P < 0.0001 were regarded as statistically significant. In addition, “ns” denoted no significant difference.

## 3 Results

### 3.1 Preparation of GMA-GEL and DA-SA

**FIGURE 2:**
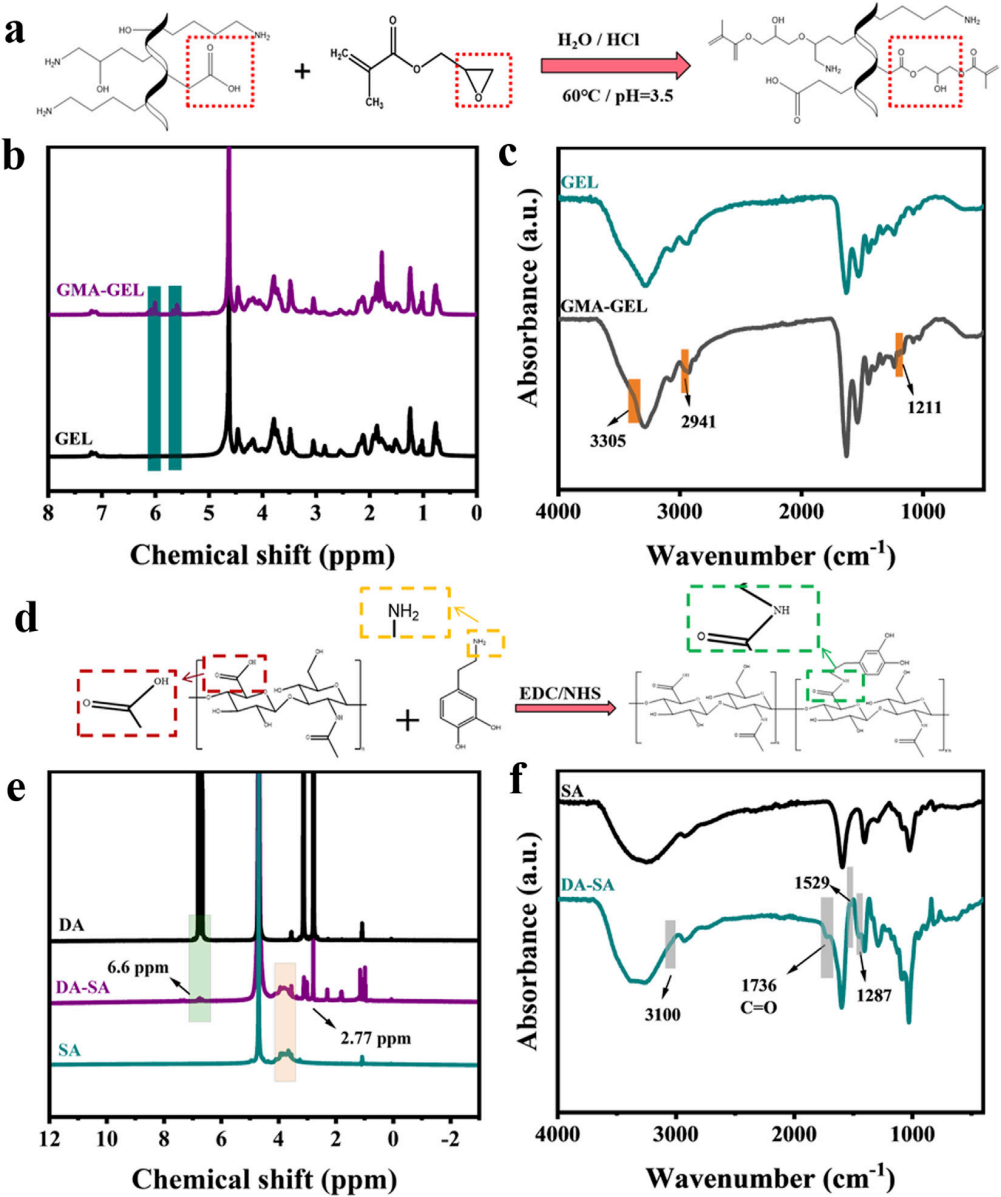
**(a)** Principles of GMA and GEL reactions; **(b)** 1H NMR spectra of gelatin before and after grafting of GMA; **(c)** FT-IR spectra of gelatin before and after grafting of GMA; **(d)** Principles of DA and SA reactions; **(e)** 1H NMR spectra of SA before and after grafting of DA; **(f)** FT-IR spectra of SA before and after grafting of DA.

Principles of GMA and GEL reactions as shown in Figure 2A. Figure 2B showed the ^1^H NMR spectra of GEL and GMA-GEL compounds. The ^1^H NMR spectra of GMA-GEL revealing the GMA grafting onto gelatin. In this spectrum, the signals observed at 5.4 and 5.8 ppm are attributed to the methacrylate vinyl proton C=C bond vibrations. The FT-IR spectra of GMA-GEL and GEL were showed in Figure 2C. Compared with the FT-IR spectrum of unmodified gelatin, the product GMA-GEL showed enhanced characteristic peaks at 1,211 cm^−1^, indicating that GMA was successfully grafted. The FT-IR spectra indicated that the stretching vibration peak of the -OH group was observed at 3,305 cm^−1^; the peak at 2,941 cm^−1^ was attributed to C-H stretching vibration; and the peak at 1,211 cm^−1^ was attributed to the ester bond formed by GMA grafting. The ^1^H NMR spectrum and FT-IR spectrum collectively demonstrated that GMA had been successfully grafted into the gelatin molecular chain.

Principles of DA and SA reactions as shown in Figure 2D. Figure 2E showed the ^1^H NMR spectra of DA, SA, and DA-SA. The ^1^H NMR spectra of DA-SA revealed that the peak at 6.6 ppm specifically corresponded to the proton -CH on the catechol ring, and the -NH group near the catechol ring was observed around 2.77 ppm, thus confirming the successful grafting of the catechol motif. Figure 2F showed the FT-IR spectra of DA, SA, and DA-SA. In the FT-IR spectrum of DA-SA, two characteristic peaks were observed at 1,287 and 1,529 cm^−1^, which were attributed to the amide III band (C-N stretching vibration) and amide II band (N-H bending vibration), respectively. Additionally, the amide I band was observed at 1,736 cm^−1^. The presence of the amide group (-CONH-) at 3,100 cm^−1^ indicated the successful grafting of DA. Both ^1^H NMR spectra and FT-IR spectra were consistent in showing that DA was successfully grafted into the SA molecular chain.

### 3.2 Preparation and characterization of PDGA hydrogel

In the process of soaking PAAM/DA-SA/GMA-GEL, PAAM, PAAM/DA-SA, PAAM/GMA-GEL, DA-SA, GMA-GEL, and DA-SA/GMA-GEL in deionized water for 3 days to remove unreacted monomers and initiators.

**FIGURE 3:**
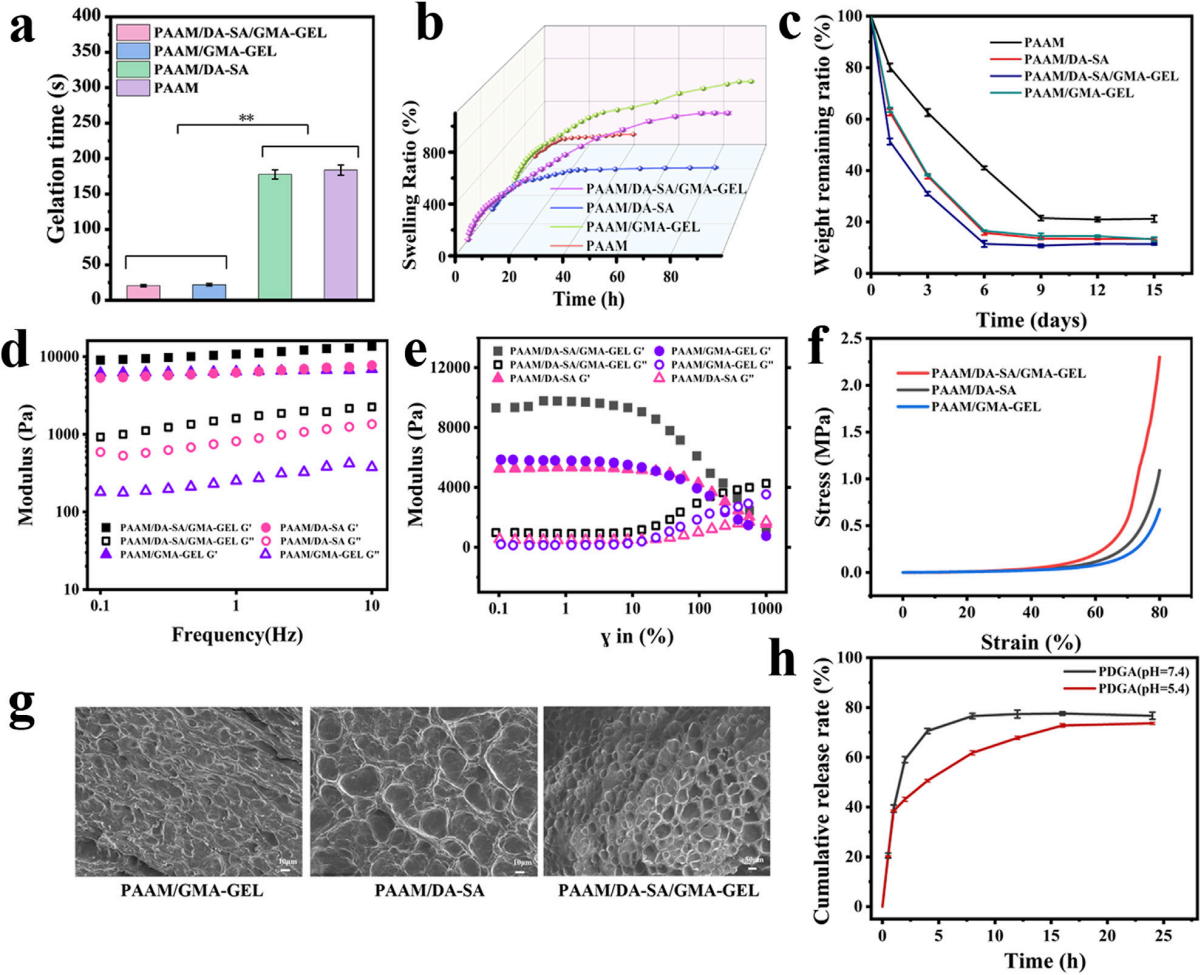
**(a)** Gelation times; **(b)** Swelling performance curves for hydrogels with various compositions; **(c)** Degradation performance curve of different component gels; **(d)** Rheological characteristics of hydrogels at angular frequencies ranging from 0 to 10 rad/s; **(e)** Rheological behavior of hydrogels under varying strains; **(f)** Compressive properties of hydrogels with diverse compositions; **(g)** SEM of hydrogels; **(h)** Rate of drug release at different pH.

The gelation time of the hydrogels was evaluated by the time of irradiation under 365 nm UV light to turn the gel precursor solution into a gel, and the gelling time of PAAM/DA-SA/GMA-GEL and PDGA hydrogels was about 20 s. As shown in Figure 3A. The UV irradiation time was safe for biological tissues and was favorable for *in vitro* applications (Xu et al., 2022).

During the wound healing process, tissue fluid may persist in exuding. Hydrogel dressings possessing excellent swelling capabilities can efficiently absorb these exudates and create a moist microenvironment for the wound. This moist microenvironment facilitates cell proliferation and differentiation, thereby expediting the wound healing process. Therefore, the swelling properties of these hydrogels were tested. As shown in Figure 3B. The ideal dressing needs to have excellent swelling properties, but PAAM does not have this property. By incorporating DA-SA and GMA-GEL, on one hand, the introduction of additional hydrophilic groups, such as hydroxyl and amino functionalities, facilitates the formation of interactions with water molecules, including hydrogen bonding, thereby enhancing the water absorption capacity and swelling behavior of the hydrogel. On the other hand, the integration of PAAM, DA-SA, and GMA-GEL results in the formation of double cross-linked structure, enabling more efficient retention of water, thereby further enhancing the swelling characteristics of the hydrogel.

The degradation properties of the hydrogels were investigated by simulating a physiological environment at 37°C PBS buffer for evaluating the degradation properties of the hydrogels After 3 days of hydrogel immersion in PBS, the residual weight ratio of the PAAM hydrogel was 62% of the initial weight, whereas that of the PAAM/DA-SA, PAAM/DA-SA/GMA-GEL and PAAM/GMA-GEL hydrogels was about 30% of the initial weight. Residual ratio was about 30% of the initial weight. After the PAAM hydrogels reached the degradation equilibrium at day 9, the weight remaining was maintained at about 20%, while the PAAM/DA-SA, PAAM/DA-SA/GMA-GEL and PAAM/GMA-GEL hydrogels reached the degradation equilibrium at day 6, indicating that the PAAM/DA-SA/GMA-GEL hydrogels had a tunable degradation ratio by the introduction of DA-SA and GMA-GEL. hydrogels with adjustable degradation rates by the introduction of DA-SA and GMA-GEL.

The rheological properties of the hydrogels were assessed, with the temperature set at 37°C to mimic the body temperature of human skin (Fares et al., 2024). The storage modulus (G′) and loss modulus (G'') of different hydrogels were tested at a fixed frequency of 10 rad s^−1^. It was found that the storage modulus (G′) was higher than the loss modulus (G'') for all hydrogels, implying that they were stable (Figure 3D). As shown in Figure 3E, no significant difference in fracture strain of hydrogels.

The compression properties of the different component hydrogels are shown in Figure 3F. Compared to the PAAM/GMA-GEL group and the PAAM/DA-SA group, the addition of DA-SA and GMA-GEL to the hydrogels resulted in an increase in compressive strength. The high molecular mass of DA-SA and the presence of more polar hydroxyl groups enhanced the entanglement between polymer chains through hydrogen bonding. This resulted in a denser structure of the hydrogel, subsequently increasing its compressive strength. The incorporation of GMA-GEL via photo-crosslinking and chemical crosslinking contributed to a denser gel structure. Collectively, these results suggested that PAAM/DA-SA/GMA-GEL hydrogels exhibited good energy dissipation capacity under external forces, owing to their non-covalent cross-linking network.

During the synthesis process of PAAM/DA-SA/GMA-GEL hydrogels, the polymer chains undergo both chemical and photocross-linking to establish a dual cross-linked network architecture. The resultant uniform morphology and interconnected pore structure empower the hydrogel to preserve its structural robustness and coherence under external stress (Figure 3G).

The PDGA (pH = 7.4) hydrogel had reached drug release equilibrium at 8 h with a drug release rate of 76.68%, whereas the PDGA (pH = 5.4) hydrogel effectively reduced the drug release efficiency and reached drug release equilibrium at 24 h with a release rate of 73.65%. The PDGA hydrogel at pH = 5.4 helped in the slow release of the drug (Figure 3H).

### 3.3 Evaluation of hydrogel biocompatibility

**FIGURE 4:**
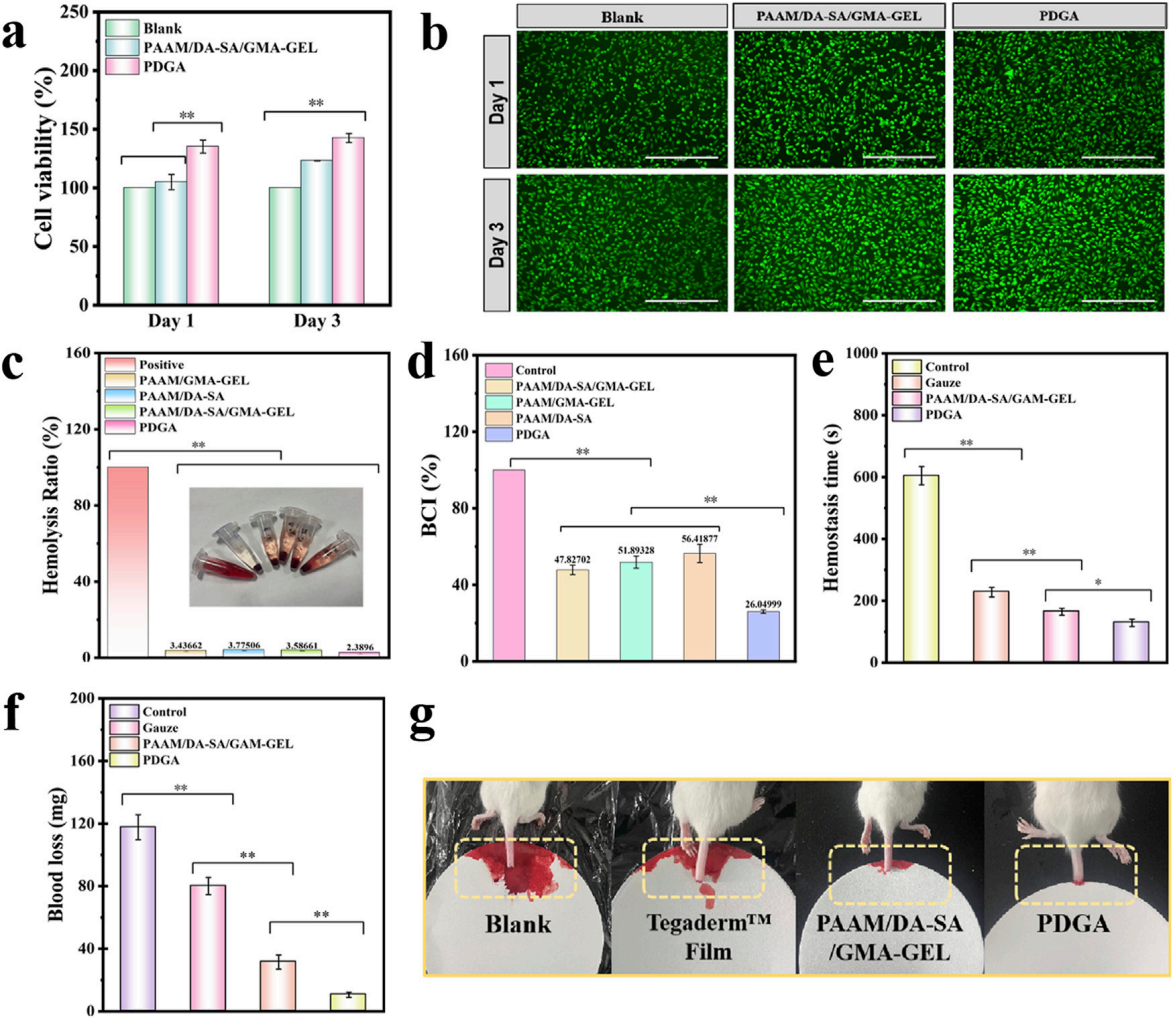
**(a)** Cell compatibility assessment of the PDGA and PAAM/DA-SA/GMA-GEL hydrogel; **(b)** LIVE/DEAD staining of L929 cells after contacted with the hydrogels for 1 d and 3d; Scale bar: 400 μm; **(c)** Hemolysis rate (%) of various hydrogels; **(d)** Whole blood clotting time assay; **(e)** Blood loss time in the mouse-tail amputation model; **(f)** Quantitative results of blood loss in the mouse-tail amputation model (n = 3); **(g)** Schematic diagram of the mouse-tail amputation model. Statistical significance *P < 0.05, **P < 0.01.

The cytocompatibility of PAAM/DA-SA/GMA-GEL and PDGA hydrogels was assessed using the cell viability of L929 cells. Cytotoxicity of the aforementioned hydrogels was determined using the leachate method. A significant increase in cell viability was observed within 3 days, suggesting robust cell growth during the experimental period (Figure 4A). On the day 3, the cell viability of PDGA hydrogel was significantly higher than that of PAAM/DA-SA/GMA-GEL and the control group (P < 0.01). In addition, almost all L929 cells showed a pike-shaped morphology and were stained green (live cells), and only a few red (dead cells) cells were observed in live/dead viability staining, suggesting good cytocompatibility of the immersed hydrogel (Figure 4B). The results demonstrate that the hydrogel exhibits good cytocompatibility with no apparent cytotoxicity, indicating its potential as an alternative material for clinical dressings.

Good blood compatibility is crucial for the application of biomaterials. An *in vitro* hemolysis assessment was conducted to evaluate the blood compatibility of PDGA hydrogels. After a 1-h incubation in a simulated physiological environment *in vitro*, qualitative observations were made on the visual appearance of the four hydrogel groups, along with the positive control group, as shown in Figure 4C. The hydrogels exhibited a Quantitative analysis revealed that the hemolysis rates for all hydrogels were below 5%. These results indicate good blood compatibility for these hydrogels.

The human skin and tissues are densely populated with blood vessels. Upon injury or trauma caused by external forces, these blood vessels may rupture, resulting in blood loss. Therefore, achieving hemostasis is a crucial initial step in the wound healing process. An *in vitro* whole-blood coagulation assessment is a widely used method to evaluate the coagulation efficacy of hemostatic hydrogels (Bao et al., 2022). A lower Blood Coagulation Index (BCI) signifies higher coagulation efficiency. Medical gauze was selected as the control material for comparison with the hydrogels. Following incubation of various hydrogels with blood at 37 °C for 10 min, the results revealed that the BCI values for all hydrogel groups were significantly lower than that of the gauze control (P < 0.05) (Figure 4D).

Due to the good blood compatibility and blood-clotting index of PDGA hydrogel, its hemostatic performance was further evaluated using a mouse-tail amputation model (Figures 4E–G). The hemostatic performance of the PDGA hydrogel was tested in the mouse-tail amputation model, resulting in a significant reduction in blood loss (P < 0.01) and bleeding time. Compared to the control group, rat tails treated with PDGA hydrogel exhibited a 91.4% reduction in blood loss and a 78.8% decrease in bleeding time. The *in vivo* hemostatic effect of the PDGA hydrogel was primarily attributed to the angelica polysaccharides within the hydrogel interacted with integrins on the platelet surface, leading to increased platelet adhesion, activation, and aggregation, thereby enhancing hemostasis.

### 3.4 *In vivo* wound healing in full-thickness skin defect model

**FIGURE 5:**
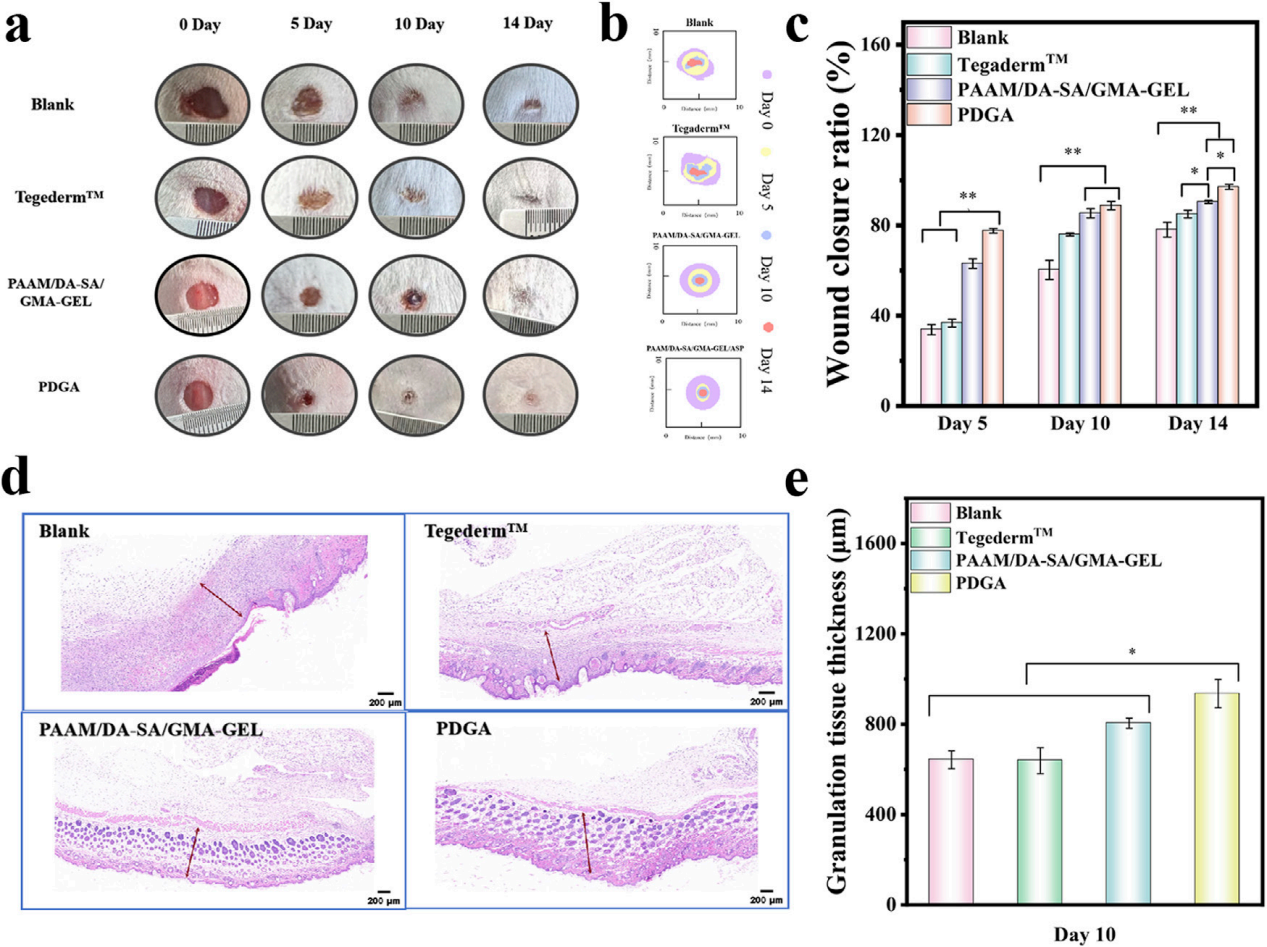
**(a)** Photographs of the wound healing site captured on the 5th, 10th, and 14th days; **(b)** Schematic illustration of the wound healing process on days 5, 10, and 14; **(c)** Statistical analysis of wound closure ratio (n = 3); **(d)** Micrographs of regenerating granulation tissue on the 10th day (granulation tissue indicated by red arrows), scale bar: 200 μm; **(e)** Thickness measurements of the regenerated granulation tissue on the 10th day (n = 3). Statistical significance *P < 0.05, **P < 0.01.

To assess the efficacy of these hydrogels as wound dressings, a mouse model of dorsal skin defect was established. Two control groups were established using Blank and Tegaderm™ films. The wound area in all four groups exhibited a gradual decrease over the course of 5, 10, and 14 days (Figures 5A–C). Following 5 days of treatment, the wound areas in the PAAM/DA-SA/GMA-GEL and PDGA hydrogel groups were significantly smaller compared to those in the commercial Membrane and Blank groups (Figure 5C). The wound closure ratio of the PDGA hydrogel group was approximately 44% higher than that of the Blank group (P < 0.01), indicating the most effective wound healing. The wound closure ratios of the PAAM/DA-SA/GMA-GEL and PDGA hydrogel groups were comparable and significantly higher than those of the Blank and Tegaderm™ Film groups. After 10 days of treatment, the wound closure ratios of the PAAM/DA-SA/GMA-GEL and PDGA hydrogel groups were 85% and 88%, respectively. These results suggest that the wound healing efficacy of these hydrogels exceeded that of both the Tegaderm™ Film and Blank groups. Furthermore, the wound area recovery of the PAAM/DA-SA/GMA-GEL and PDGA hydrogel groups was significantly greater than that of the Tegaderm™ Film and Blank groups (P < 0.01). On day 14, the wound in the PDGA group achieved near-complete healing, with a wound area recovery exceeding 97%.

Granulation tissue is a newly formed, capillary-rich fibrous connective tissue that plays a key role in the healing process after tissue injury (Clark, 1985). Therefore, the stage of wound healing was assessed by measuring the thickness of the granulation tissue. As shown in Figures 5D, E, after 10 days of healing, the Blank and Tegaderm™ Film groups exhibited a thinner granulation tissue thickness (642.8 μm and 638.5 μm, respectively). The granulation tissue thickness of the PAAM/DA-SA/GMA-GEL and PDGA hydrogels were 804.4 μm and 935 μm, respectively. The latter was significantly thicker than that of the other groups (P < 0.05). These results indicate that the PDGA hydrogel has a more favorable effect on wound repair.

### 3.5 Histomorphological evaluation

**FIGURE 6:**
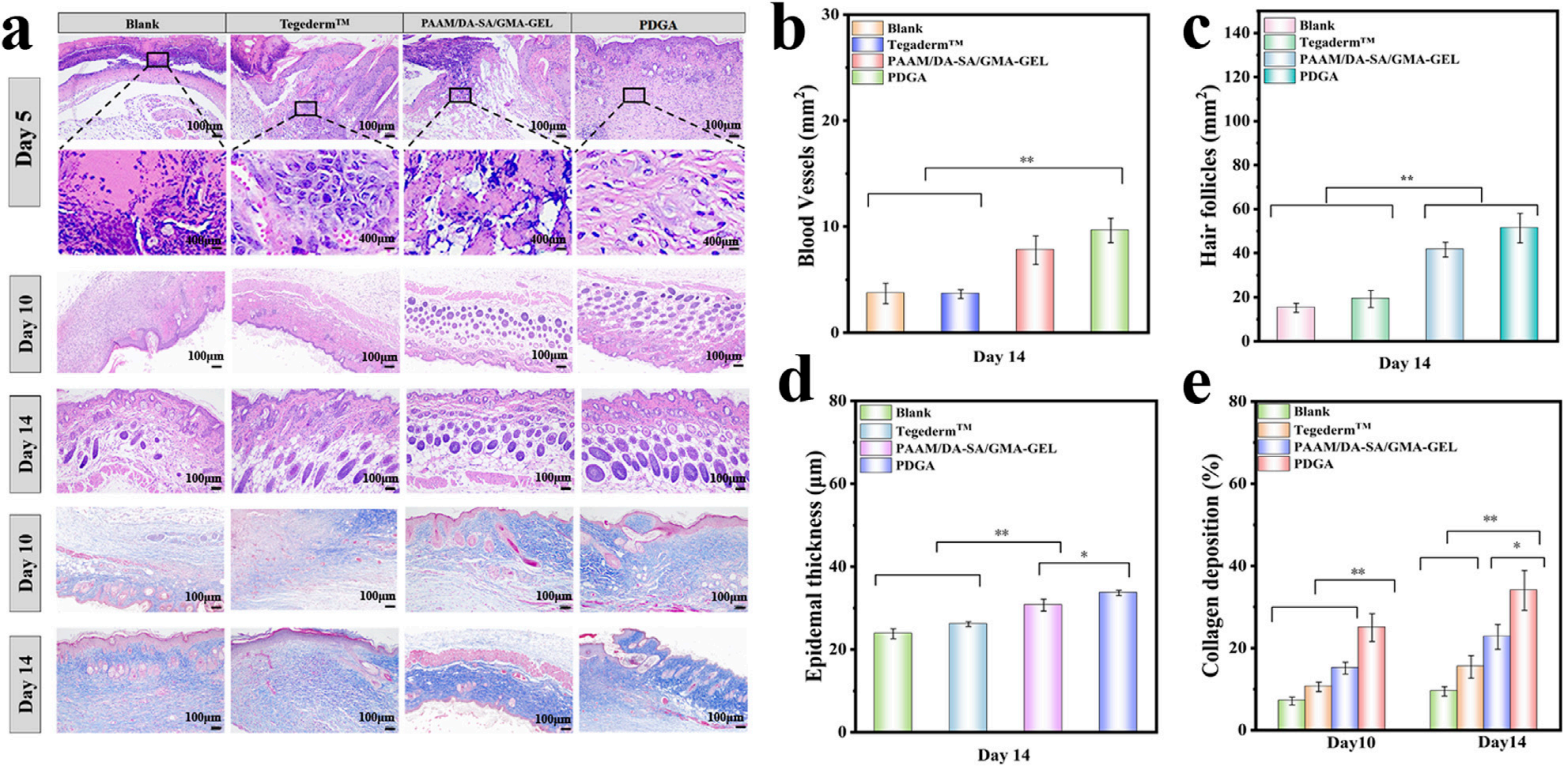
**(a)** Morphological results of the wound healing site after treatment for 5, 10, and 14 days, with Masson’s trichrome staining at the wound site on the 10th and 14th days; **(b)** Regeneration of blood vessels on the 14th day post-treatment; **(c)** Newly formed hair follicles on the 14th day post-treatment; **(d)** Epidermal thickness for different groups on the 14th day post-treatment; **(e)** Collagen deposition. Statistical significance *P < 0.05, **P < 0.01.

Wound healing is a continuous process that encompasses wound contraction, granulation tissue formation, and regeneration of the epidermis and other tissues (Wang et al., 2023). Hematoxylin and eosin-stained sections (H&E staining) were used to assess the wound healing effect on days 5, 10, and 14 (Shamloo et al., 2018). On the fifth day of wound healing, HE staining revealed no significant inflammatory reaction in any of the groups. However, compared to the two hydrogel groups, more inflammatory cells were observed at the wound site in the blank and Tegaderm™ groups (Figure 6A). Furthermore, the PDGA hydrogel group exhibited relatively fewer inflammatory cells and a higher presence of fibroblasts compared to the PAAM/DA-SA/GMA-GEL hydrogel group. These results indicate that the PDGA hydrogels did not elicit an increased foreign body response. Neovascularization is crucial for repairing the blood microcirculatory system of subcutaneous damaged tissues, thereby providing essential nutrients. As shown in Figure 6B, both the PAAM/DA-SA/GMA-GEL and PDGA hydrogel groups displayed a greater number of blood vessels compared to the blank and Tegaderm™ groups (P < 0.05). Additionally, some hair follicles formed in the PDGA group (Figure 6C). On day 14, although relatively intact skin had grown in the blank and Tegaderm™ Film groups, few hair follicles were observed (Figure 6A). In contrast, the wounds treated with hydrogels were covered with epithelial tissues that more closely resembled normal skin. The increased number of skin follicles and blood vessels in the PAAM/DA-SA/GMA-GEL and PDGA hydrogel groups was also significantly different from that in the blank and Tegaderm™ groups (P < 0.05). Notably, both the PAAM/DA-SA/GMA-GEL group and the PDGA group exhibited nearly intact epidermis recovery (Figure 6D). These results indicate that the hydrogel was beneficial to ECM remodeling and tissue regeneration. In addition, ASP can significantly stimulate the proliferation of hematopoietic stem cells and promote the synthesis of hemoglobin and red blood cells (Bai et al., 2018; Zhang et al., 2019; Chi et al., 2022; Guo et al., 2024).

Collagen deposition plays a critical role in tissue remodeling during wound healing. As shown in Figure 6E, the amount of collagen deposition in the PDGA hydrogel group was significantly higher than in the PAAM/DA-SA/GMA-GEL hydrogel group on the 10th day (P < 0.01). On the 14th day, the collagen volume ratio of the PDGA hydrogel group was still significantly higher than that in the Tegaderm™ group and Blank group (P < 0.01). The collagen volume ratio at the wound site continued to increase over the 14 days of treatment. The hydrogel group exhibited a better collagen volume ratio throughout the repair process compared to the Tegaderm™ and blank groups. Masson trichrome staining of tissue sections was performed on days 10 and 14 to qualitatively observe and quantitatively analyze collagen deposition (Figure 6A). Collagen during wound recovery was stained blue. The PDGA group showed the best collagen regeneration compared to the other hydrogel groups, as well as the Tegaderm™ membrane and blank groups. Thus, these results demonstrate that PDGA hydrogel can effectively promote collagen deposition.

### 3.6 The expression of CD31 during wound healing

**FIGURE 7:**
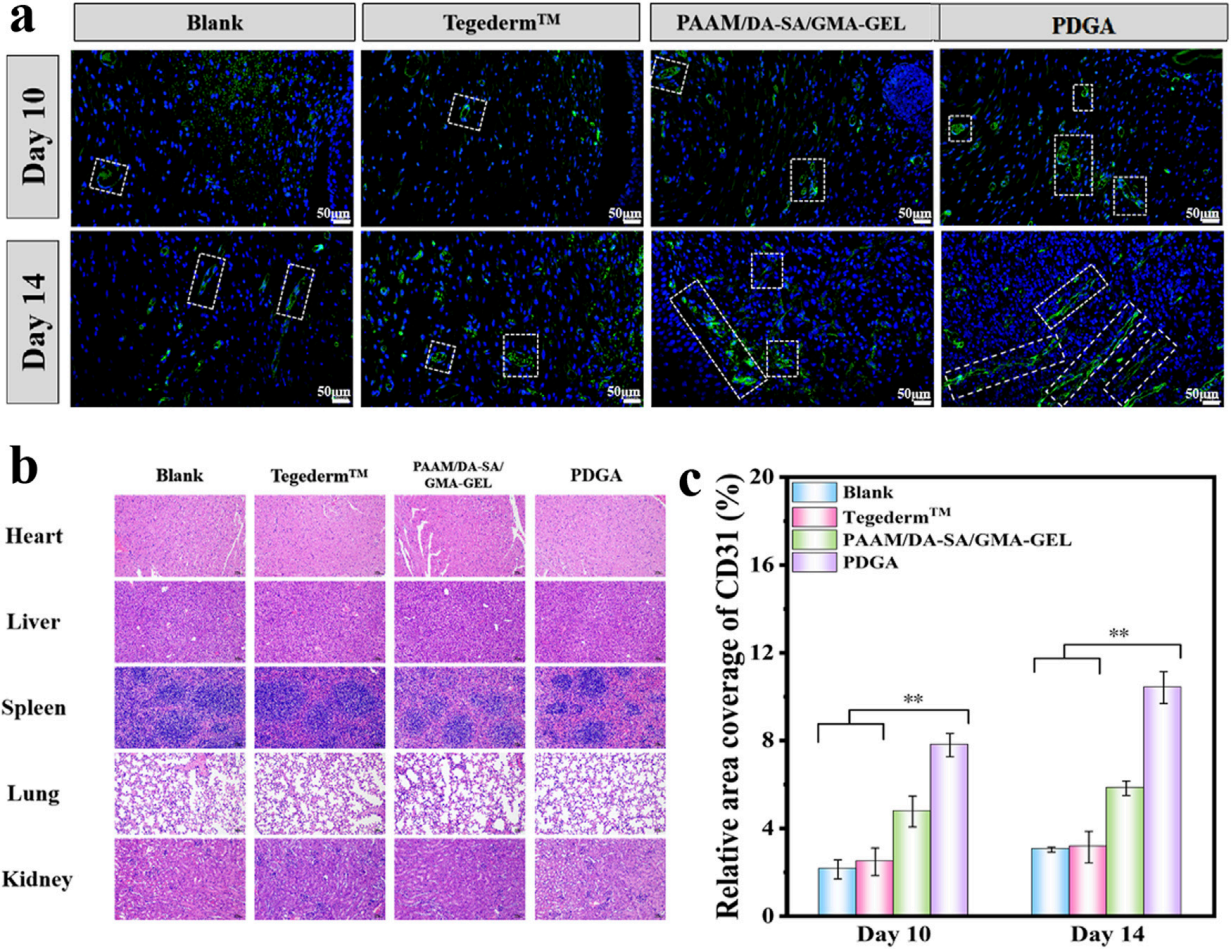
**(a)** Expression of CD31 on 10 and 14 days; White line indicate the expression of CD31; **(b)** HE staining results of liver, spleen, lungs, and kidneys in each group 14 days after surgery; **(c)** With n = 3, the CD31 relative area ratio was calculated. Statistical significance *P < 0.05, **P < 0.01.

CD31 is a member of the vascular endothelial cell markers and is mainly expressed on the surface of endothelial cells, B-lymphocytes, platelets, granulocytes, and certain T cells (Yang et al., 2020; Guo et al., 2022; Liu et al., 2024). Consequently, we selected CD31 as an indicator for evaluating wound dressings to promote angiogenesis during wound healing. The immunofluorescence staining of CD31 and quantitative results were shown in Figures 7A, C. Compared with Blank, Tegaderm™ Film, and PAAM/DA-SA/GMA-GEL, the wound site treated with the PDGA hydrogel showed more CD31 expression (P < 0.01) on the 10th day. In addition, on the 14th day, the expression levels of CD31 in the PDGA group were significantly higher than those of other groups (P < 0.01). In conclusion, the PDGA hydrogel significantly promotes wound healing by enhancing CD31 expression, collagen deposition, angiogenesis, and hair follicle generation. Compared to the blank group and Tegaderm™, the PDGA hydrogel demonstrates superior repair efficacy.

Pathological examination was conducted on the hearts, liver, spleen, lungs, and kidneys of mice in the blank group, Tegaderm™ group, PAAM/DA-SA/GMA-GEL group, and PDGA group 14 days postsurgery. No significant abnormalities were found, thus indicating that PDGA hydrogel did not cause damage to any organ tissues.

## 4 Discussion

PDGA hydrogel benefits from the rapid response of the photocross-linking process, achieving rapid curing, which is particularly important for emergency hemostasis scenarios and bleeding situations can be rapidly control. At the same time, its low initial crosslinking degree gives the material good shape adaptability, enabling PDGA hydrogel to closely fit the surface of various complex wounds, effectively forming a protective barrier, blocking the invasion of bacteria and microorganisms, and reducing the risk of infection. Over time, the chemical cross-linking mechanism is gradually activated and strengthened, enhancing the mechanical strength and adhesion properties of the PDGA hydrogel, ensuring the durable and stable attachment of the hydrogel at the wound site, and providing a more reliable support for the wound healing.

## 5 Conclusion

A hydrogel dressing (PDGA) with *in situ* formation properties which has been applied for rapid hemostasis, wound healing and repair was developed. The dual-crosslinked structure of PDGA hydrogels was constructed through the combined action of chemical crosslinking initiated by PAAM radical polymerization and photocrosslinking mediated by GMA-GEL radical polymerization. The hydrogel facilitates the sustained release of ASP. PDGA hydrogel can significantly improve the speed and quality of wound healing by promoting cell proliferation, platelet adhesion and aggregation, collagen deposition, vascularization and folliculogenesis. The *in situ* tissue engineering of the PDGA hydrogel allows the material to have good shape adaptability to closely fit the surface of a variety of complex wounds, effectively forming a protective barrier that stops the invasion of bacteria and microorganisms and reduces the risk of infection while also allowing for rapid control of bleeding situations. This is particularly important in emergency hemostasis scenarios, where there is a strong need for fast, effective and safe wound treatment strategies in the operating room environment. We anticipate that this study will provide a scientific basis for the application of hydrogels in wound healing and provide new ideas for the development of novel wound treatment strategies.

## Data Availability

The original contributions presented in the study are included in the article/Supplementary Material, further inquiries can be directed to the corresponding author.

## References

[B1] Agarwal R. Niezgoda J. Niezgoda J. Madetipati N. Gopalakrishnan S. (2022). Advances in hemostatic wound dressings: clinical implications and insight. Adv. Skin. Wound Care 35 (2), 113–121. 10.1097/01.ASW.0000790488.72494.57 34516437

[B2] Bai Z. Dan W. Yu G. Wang Y. Chen Y. Huang Y. (2018). Tough and tissue-adhesive polyacrylamide/collagen hydrogel with dopamine-grafted oxidized sodium alginate as crosslinker for cutaneous wound healing. RSC Adv. 8 (73), 42123–42132. 10.1039/c8ra07697a 35558764 PMC9092085

[B3] Bao L. Li C. Tang M. Chen L. Hong F. F. (2022). Potential of a composite conduit with bacterial nanocellulose and fish gelatin for application as small-diameter artificial blood vessel. Polym. (Basel) 14 (20), 4367. 10.3390/polym14204367 PMC9610583 36297946

[B4] Bi S.-J. Fu R.-J. Li J.-J. Chen Y.-Y. Tang Y.-P. (2021). The bioactivities and potential clinical values of angelica sinensis polysaccharides. Nat. Product. Commun. 16 (3). 10.1177/1934578x21997321

[B5] Brumberg V. Astrelina T. Malivanova T. Samoilov A. (2021). Modern wound dressings: hydrogel dressings. Biomedicines 9 (9), 1235. 10.3390/biomedicines9091235 34572421 PMC8472341

[B6] Chen S. Wang Y. Lai J. Tan S. Wang M. (2023). Structure and properties of gelatin methacryloyl (GelMA) synthesized in different reaction systems. Biomacromolecules 24 (6), 2928–2941. 10.1021/acs.biomac.3c00302 37212876

[B7] Chi J. Li A. Zou M. Wang S. Liu C. Hu R. (2022). Novel dopamine-modified oxidized sodium alginate hydrogels promote angiogenesis and accelerate healing of chronic diabetic wounds. Int. J. Biol. Macromol. 203, 492–504. 10.1016/j.ijbiomac.2022.01.153 35101479

[B8] Chou P. Y. Chen S. H. Chen C. H. Chen S. H. Fong Y. T. Chen J. P. (2017). Thermo-responsive *in-situ* forming hydrogels as barriers to prevent post-operative peritendinous adhesion. Acta Biomater. 63, 85–95. 10.1016/j.actbio.2017.09.010 28919215

[B9] Ciolacu D. E. Nicu R. Suflet D. M. Rusu D. Darie-Nita R. N. Simionescu N. (2023). Multifunctional hydrogels based on cellulose and modified lignin for advanced wounds management. Pharmaceutics 15 (11), 2588. 10.3390/pharmaceutics15112588 38004566 PMC10674243

[B10] Clark R. A. (1985). Cutaneous tissue repair: basic biologic considerations. I. J. Am. Acad. Dermatol 13 (5 Pt 1), 701–725. 10.1016/s0190-9622(85)70213-7 2416789

[B11] Del Giudice F. (2020). Simultaneous measurement of rheological properties in a microfluidic rheometer. Phys. Fluids 32 (5). 10.1063/5.0006060

[B12] Ding L. He L. Wang Y. Zhao X. Ma H. Luo Y. (2023). Research progress and challenges of composite wound dressings containing plant extracts. Cellulose 30 (18), 11297–11322. 10.1007/s10570-023-05602-0

[B13] Fang J. Li H. Wang J. Yang M. Zong Z. Zhang Y. (2019). Compression and stress relaxation characteristics of alfalfa during rotary compression. BioResources 14 (2), 3860–3872. 10.15376/biores.14.2.3860-3872

[B14] Fares M. M. Radaydeh S. K. Jabani Z. H. (2024). IPN based hydrogels for *in-vivo* wound dressings; catalytic wound healing dynamics and isothermal adsorption models. J. Photochem Photobiol. B 254, 112901. 10.1016/j.jphotobiol.2024.112901 38552571

[B15] Feng F. Zhao Z. Li J. Huang Y. Chen W. (2024). Multifunctional dressings for wound exudate management. Prog. Mater. Sci. 146, 101328. 10.1016/j.pmatsci.2024.101328

[B16] Feng Y. Qin S. Li H. Yang Y. Zheng Y. Liu H. (2023). Composite hydrogel dressings with enhanced mechanical properties and anti-inflammatory ability for effectively promoting wound repair. Int. J. Nanomedicine 18, 5183–5195. 10.2147/IJN.S411478 37720596 PMC10503508

[B17] Francesko A. Petkova P. Tzanov T. (2018). Hydrogel dressings for advanced wound management. Curr. Med. Chem. 25 (41), 5782–5797. 10.2174/0929867324666170920161246 28933299

[B18] Guo J. Wang T. Yan Z. Ji D. Li J. Pan H. (2022). Preparation and evaluation of dual drug-loaded nanofiber membranes based on coaxial electrostatic spinning technology. Int. J. Pharm. 629, 122410. 10.1016/j.ijpharm.2022.122410 36402289

[B19] Guo Y. Shao Z. Wang W. Liu H. Zhao W. Wang L. (2024). Periodontium-mimicking, multifunctional biomass-based hydrogel promotes full-course socket healing. Biomacromolecules 25 (2), 1246–1261. 10.1021/acs.biomac.3c01221 38305191

[B20] Hasani-Sadrabadi M. M. Sarrion P. Pouraghaei S. Chau Y. Ansari S. Li S. (2020). An engineered cell-laden adhesive hydrogel promotes craniofacial bone tissue regeneration in rats. Sci. Transl. Med. 12 (534), eaay6853. 10.1126/scitranslmed.aay6853 32161103

[B21] Jones J. Hampton S. (2021). Use of a superabsorbent dressing in the management of exudate in hard-to-heal wounds. Br. J. Community Nurs. 26 (Suppl. 3), S20–S29. 10.12968/bjcn.2021.26.Sup3.S20 33688756

[B22] Kamoun E. A. Kenawy E. S. Chen X. (2017). A review on polymeric hydrogel membranes for wound dressing applications: PVA-based hydrogel dressings. J. Adv. Res. 8 (3), 217–233. 10.1016/j.jare.2017.01.005 28239493 PMC5315442

[B23] Klabukov I. Shestakova V. Krasilnikova O. Smirnova A. Abramova O. Baranovskii D. (2023). Refinement of animal experiments: replacing traumatic methods of laboratory animal marking with non-invasive alternatives. Anim. (Basel) 13 (22), 3452. 10.3390/ani13223452 PMC10668729 38003070

[B24] Kowalski G. Witczak M. Kuterasinski L. (2024). Structure effects on swelling properties of hydrogels based on sodium alginate and acrylic polymers. Molecules 29 (9), 1937. 10.3390/molecules29091937 38731429 PMC11085423

[B25] Krasilnikova O. A. Baranovskii D. S. Yakimova A. O. Arguchinskaya N. Kisel A. Sosin D. (2022). Intraoperative creation of tissue-engineered grafts with minimally manipulated cells: new concept of bone tissue engineering *in situ* . Bioeng. (Basel) 9 (11), 704. 10.3390/bioengineering9110704 PMC9687730 36421105

[B26] Li J. Shen J. Zhuang B. Wei M. Liu Y. Liu D. (2022). Light-triggered on-site rapid formation of antibacterial hydrogel dressings for accelerated healing of infected wounds. Biomater. Adv. 136, 212784. 10.1016/j.bioadv.2022.212784 35929299

[B27] Liu F. Xu X. Sun T. (2024). Vascular endothelial growth factor accelerates healing of foot ulcers in diabetic rats via promoting M2 macrophage polarization. Diabet. Med. 41 (9), e15388. 10.1111/dme.15388 38934613

[B28] Nai J. Zhang C. Shao H. Li B. Li H. Gao L. (2021). Extraction, structure, pharmacological activities and drug carrier applications of Angelica sinensis polysaccharide. Int. J. Biol. Macromol. 183, 2337–2353. 10.1016/j.ijbiomac.2021.05.213 34090852

[B29] Reinhards-Hervás C. Cano A. J. Rico A. Salazar A. Rodríguez J. (2024). Fracture resistance of polyacrylamide-alginate hydrogels. Eng. Fract. Mech. 295, 109812. 10.1016/j.engfracmech.2023.109812

[B30] Shamloo A. Sarmadi M. Aghababaie Z. Vossoughi M. (2018). Accelerated full-thickness wound healing via sustained bFGF delivery based on a PVA/chitosan/gelatin hydrogel incorporating PCL microspheres. Int. J. Pharm. 537 (1-2), 278–289. 10.1016/j.ijpharm.2017.12.045 29288809

[B31] Sk M. M. Das P. Panwar A. Tan L. P. (2021). Synthesis and characterization of site selective photo-crosslinkable glycidyl methacrylate functionalized gelatin-based 3D hydrogel scaffold for liver tissue engineering. Mater Sci. Eng. C Mater Biol. Appl. 123, 111694. 10.1016/j.msec.2020.111694 33812568

[B32] Tavakoli S. Klar A. S. (2020). Advanced hydrogels as wound dressings. Biomolecules 10 (8), 1169. 10.3390/biom10081169 32796593 PMC7464761

[B33] Tavakoli S. Klar A. S. (2021). Bioengineered skin substitutes: advances and future trends. Appl. Sci. 11 (4), 1493. 10.3390/app11041493

[B34] Tian Y. Shen X. Hu T. Liang Z. Ding Y. Dai H. (2024). Structural analysis and blood-enriching effects comparison based on biological potency of Angelica sinensis polysaccharides. Front. Pharmacol. 15, 1405342. 10.3389/fphar.2024.1405342 38953103 PMC11215113

[B35] Tirey T. N. Singh A. Arango J. C. Claridge S. A. (2024). Nanoscale surface chemical patterning of soft polyacrylamide with elastic modulus similar to soft tissue. Chem. Mater. 36 (17), 8264–8273. 10.1021/acs.chemmater.4c01106 39279906 PMC11397139

[B36] Tottoli E. M. Dorati R. Genta I. Chiesa E. Pisani S. Conti B. (2020). Skin wound healing process and new emerging technologies for skin wound Care and regeneration. Pharmaceutics 12 (8), 735. 10.3390/pharmaceutics12080735 32764269 PMC7463929

[B37] Villa C. Cuna F. Grignani E. Perteghella S. Panzeri D. Caviglia D. (2024). Evaluation of the biological activity of manna exudate, from fraxinus ornus L., and its potential use as hydrogel formulation in dermatology and cosmetology. Gels 10 (6), 351. 10.3390/gels10060351 38920898 PMC11202673

[B38] Wang J. Lian P. Yu Q. Wei J. Kang W. Y. (2017). Purification, characterization and procoagulant activity of polysaccharides from Angelica dahurice roots. Chem. Cent. J. 11, 17. 10.1186/s13065-017-0243-y 28246546 PMC5307400

[B39] Wang L. Zhou M. Xu T. Zhang X. (2022a). Multifunctional hydrogel as wound dressing for intelligent wound monitoring. Chem. Eng. J. 433, 134625. 10.1016/j.cej.2022.134625

[B40] Wang Y. Feng Y. Yan J. Han X. Song P. Wu Y. (2023). Spiky surface topography of heterostructured nanoparticles for programmable acceleration of multistage wound healing. Mater. Today Nano 23, 100351. 10.1016/j.mtnano.2023.100351

[B41] Wang Y. Xiao D. Quan L. Chai H. Sui X. Wang B. (2022b). Mussel-inspired adhesive gelatin-polyacrylamide hydrogel wound dressing loaded with tetracycline hydrochloride to enhance complete skin regeneration. Soft Matter 18 (3), 662–674. 10.1039/d1sm01373d 34935829

[B42] Xu Y. Chen H. Fang Y. Wu J. (2022). Hydrogel combined with phototherapy in wound healing. Adv. Healthc. Mater 11 (16), e2200494. 10.1002/adhm.202200494 35751637

[B43] Yang M. Li C. J. Xiao Y. Guo Q. Huang Y. Su T. (2020). Ophiopogonin D promotes bone regeneration by stimulating CD31(hi) EMCN(hi) vessel formation. Cell Prolif. 53 (3), e12784. 10.1111/cpr.12784 32080957 PMC7106967

[B44] Zangeneh M. M. Saneei S. Zangeneh A. Toushmalani R. Haddadi A. Almasi M. (2019). Preparation, characterization, and evaluation of cytotoxicity, antioxidant, cutaneous wound healing, antibacterial, and antifungal effects of gold nanoparticles using the aqueous extract of *Falcaria vulgaris* leaves. Appl. Organomet. Chem. 33 (11). 10.1002/aoc.5216

[B45] Zhang H. Y. Wang K. T. Zhang Y. Cui Y. L. Wang Q. (2023). A self-healing hydrogel wound dressing based on oxidized Bletilla striata polysaccharide and cationic gelatin for skin trauma treatment. Int. J. Biol. Macromol. 253 (Pt 6), 127189. 10.1016/j.ijbiomac.2023.127189 37783245

[B46] Zhang J. Zhang S. Liu C. Lu Z. Li M. Hurren C. (2024). Photopolymerized multifunctional sodium alginate-based hydrogel for antibacterial and coagulation dressings. Int. J. Biol. Macromol. 260 (Pt 2), 129428. 10.1016/j.ijbiomac.2024.129428 38232887

[B47] Zhang W. Zhu J. H. Xu H. Huang X. P. Liu X. D. Deng C. Q. (2019). Five active components compatibility of astragali radix and angelicae sinensis radix protect hematopoietic function against cyclophosphamide-induced injury in mice and t-BHP-induced injury in HSCs. Front. Pharmacol. 10, 936. 10.3389/fphar.2019.00936 31551766 PMC6735167

[B48] Zhao P. Guo Z. Wang H. Zhou B. Huang F. Dong S. (2023). A multi-crosslinking strategy of organic and inorganic compound bio-adhesive polysaccharide-based hydrogel for wound hemostasis. Biomater. Adv. 152, 213481. 10.1016/j.bioadv.2023.213481 37307771

