# Supplementary material for: Rapid in situ formation of a double cross-linked network hydrogels for wound healing promotion
Source: Front Pharmacol. 2025 Mar 18;16:1562264. doi: 10.3389/fphar.2025.1562264 (PMC11959063; doi:10.3389/fphar.2025.1562264)
Supplement: Supplementary file 1 [file DataSheet1.docx]

Supplementary Material

# Supplementary Data

## Synthesis of GMA-GEL

The sample was prepared at pH 3.5 according to the following method: 5g of gelatin was dissolved in 50 mL of buffer at 50 °C for more than 30 min at a stirring speed of 300 rpm. After the mixture was completely dissolved, a 1M HCl solution was added drop-by-drop to adjust the pH to 3.5. GMA was then added drop-by-drop to the solution, and the -COOH and -OH groups in the gelatin were reacted with GMA-GEL via an epoxide ring opening mechanism by continuous vigorous stirring at 60 °C for 24 h. Next, the solution was removed and cooled to room temperature and precipitated with ethanol for 3-5 h. The samples were dissolved in ultrapure water and dialyzed at 37 °C for 3 days, then freeze-dried. The sample was labeled as GMA-GEL-3.5 and stored at -20 °C.

## Synthesis of DA-SA

Preparation of DA-SA at pH 6: First, 0.75% sodium alginate was dissolved in deionized (DI) water. Then, a 1M HCl solution was added dropwise to the solution to adjust the pH to 6. Subsequently, EDC and NHS were dissolved in the adjusted solution and stirred at 450 rpm for more than 30 min. DA was then dissolved in this mixed solution. The mixture was prepared in the molar ratio of NHS: EDC: DA = 1.2:3:1, stirred for an additional 3 h, and then dialyzed with PBS for 3 days. The samples were labeled as DA-SA-6 and stored at -20 °C.

## Characterization

The ¹H nuclear magnetic resonance (¹H NMR) spectra of GMA-GEL and DA-SA were recorded using a Varian 500 NMR spectrometer (Varian, USA). Fourier Transform Infrared Spectroscopy (FTIR, Nexus, USA) was used to examine the functional group changes of GMA-GEL and DA-SA. Samples were analyzed in the range of 500–4000 cm⁻¹ with a resolution of 2 cm⁻¹ and an average of 32 scans.

The morphology of hydrogels was examined by a field emission scanning electron microscope (FESEM; FEI Quanta FEG 250) after all the hydrogel samples were freeze-dried and coated with a thin layer of gold.

## Swelling test

The swelling test was used to determine the swelling rate (%) (Q) and stability of the hydrogels. After freeze-drying the gel, the dry mass (W_0_) of the hydrogel was measured for a predetermined period of time, and the hydrogel sample was removed from the phosphate buffered saline (PBS) solution. Its wet weight (W_t_) was determined after absorbing excess water from the surface with filter paper. The weights were recorded every 0.5 h for the first 2 h, every 3 h for the next 9 h, and every 12 h thereafter.

The Q at different times was calculated using equation (1)

Q= (1)

Where W_t_ represents the mass (in grams) of the sample after being immersed in the solution for various time intervals, and W_0_ denotes the dry mass (in grams) of the sample.

## Rheological and mechanical test

A TA rheometer (DHR-2) was utilized to test the rheological properties of these hydrogels. A 500 μL hydrogel disk was placed between the parallel plates of 20 mm diameter. Under a constant frequency of 1 Hz and a strain of 1%, the time sweep tests were performed at 37 °C.

## Hemolytic activity test of the hydrogel

Hematocompatibility refers to the ability and performance of a material to interact with blood, without causing coagulation or thrombosis, and without impairing the composition and function of blood when in direct contact with it. Anticoagulated whole blood was made by mixing fresh mouse eye blood with 3.8% sodium citrate (1:9 v/v). The erythrocytes were then collected by centrifugation (2000 rpm, 3 min) and washed three times with PBS (2000 rpm, 3 min). The obtained erythrocytes were resuspended in PBS to prepare a 5% volume fraction erythrocyte suspension. A 50 mg lyophilized hydrogel was taken, ground into a powder, and placed in a 1.5 mL centrifuge tube. The tubes were preheated in a water bath shaker at 37 °C for 10 min. Then, 1 mL of erythrocyte suspension was added. After incubation for 1 h, centrifugation was performed at 1500 rpm for 5 min. The absorbance value (540 nm) of the supernatant was determined using a spectrophotometer (n=3). The supernatant was separated from 1 mL of erythrocyte suspension using centrifugation, and then 1 mL of PBS was added as a negative control and 1 mL of deionized water was added as a positive control.

Hemolysis rate was calculated by equation (2)

Hemolysis Ratio (%)=(2)

Where A_t_ denotes the absorbance value of the sample measured at 540nm, A_nc_ represents the absorbance value of the negative control recorded at 540 nm, and A_pc_ indicates the absorbance value of the positive control obtained at 540 nm.

## In vitro whole blood-clotting test

The hydrogel was fabricated into a cylindrical shape with dimensions of 8 mm in diameter and 8 mm in height. Subsequently, 50 μL of anticoagulated whole blood (as utilized in Section 1.6) was uniformly dispensed onto the hydrogel that had been pre-warmed to 37 °C for a duration of 15 min. Thereafter, 5 μL of 0.1 M CaCl_2_ solution was introduced to the blood prior to incubation at 37 °C for a period of 5 min. Following incubation, the dissociated erythrocytes were lysed by the gradual addition of 4 mL of ultrapure water, and subsequently, the absorbance value of the supernatant was measured at 540 nm utilizing an enzyme marker. For the blank control, 4 mL of ultrapure water was added to 50 μL of blood.

## Hemostasis performance of hydrogels

A mouse-tail amputation model was employed using Kunming mice (female, 28-32 g) to assess the hemostatic efficacy of the PDGA hydrogel. Briefly, the mouse was anesthetized by injecting 20 wt% urethane and subsequently fixed onto a surgical corkboard. The mouse was anesthetized and subsequently secured to a surgical soft plate. Fifty percent of the mouse's tail was amputated. Following the amputation, the tail was exposed to the air for 15 seconds to assess normal blood loss. Immediately afterwards, 200 μL of the PDGA hydrogel solution was administered to the bleeding site via syringe, followed by photocross-linking. After a 10-minute period, the weight of the filter paper containing the absorbed blood was measured and compared to that of a control group. All measurements were performed in triplicate.

## Cytotoxicity evaluation of the hydrogel

The cytotoxicity of hydrogels was assessed using the leaching solution method with L929 cells. Initially, the dry hydrogels PDGA and PAAM/DA-SA/GMA-GEL were sterilized at 60°C for 12 hours. The leaching solution was sterilized by filtration through a 0.22 μm filter (Millipore) for 10 seconds. L929 cell-specific culture medium (Zhongqiao Xinzhou) was used as the complete growth medium.

The cell proliferation and viability beneath the hydrogel disks were evaluated using the Cell Counting Kit-8 (CCK-8). A series of sterilized PDGA and PAAM/DA-SA/GMA-GEL hydrogel extracts with concentrations of 1, 2.5 and 5 mg/mL, respectively, were prepared by immersing each sample in cell culture medium at 37 °C for 24 h. Subsequently, L929 cells were seeded into 96-well plates at a density of 4000 cells per well and incubated in a humidified environment at 37 °C with 5% CO₂. After the L929 cells were incubated with complete growth medium for 24 h, the medium was replaced with 200 μL of the corresponding hydrogel extracts. After being co-incubated for another 24, 48 and 72 h, the extracts were removed, and 20 μL of CCK-8 reagent in 200 μL complete growth medium was added to each well in the plate. The plate was then incubated for 1 h in a humidified incubator containing 5% CO₂ at 37 °C. Subsequently, 200 μL of the medium from each well was transferred into a 96-well plate (Costar), and the microplate reader was used to measure the fluorescence of each well to assess cell viability.

## In vivo wound healing evaluation with a full-thickness skin defect model

All the animal experiments were approved by the institutional review board of Changchun University of Chinese Medicine. Female Kunming mice weighing 28-36 g and 5-6 weeks of ages were used for studies. All mice were randomly divided into 4 groups, namely Blank control, Tegaderm^TM^, PAAM/DA-SA/GMA-GEL hydrogel, and PDGA hydrogel. Each group contained 9 mices. All mice were kept normally for 7 days before the experiment to acclimate them to the environment. All procedures were performed under aseptic conditions. After the standard anesthesia procedure with intraperitoneal injection of Urethane (20%), the skin on the back of the mouse was shaved in preparation for surgery. A circular wound 6 mm in diameter was created on the back of the mouse. After the removal of wound skin, the blank control group did not receive any treatment; the Tegaderm^TM^ group wounds were treated with 200 mL of PBS and then dressed with Tegaderm^TM^ (3M Health Care, USA); and the hydrogel group wounds were treated with 200 mL of PAAM/DA-SA/GMA-GEL hydrogel and PDGA hydrogel. Tissues were collected from 3 mices in each group on the 5th, 10th, and 14th days.

All samples were stored in 4% paraformaldehyde before analysis. The recovery process of wound regeneration was assessed by observing the wound area. Wound area was observed on days 5, 10 and 14, and mice in each group were injected intraperitoneally with polyurethane (0.3 mg/kg body weight).

For the biochemical analysis, the samples were collected on the 5th, 10th and 14th days. We performed qualitative and quantitative assessments by HE staining, Masson staining, and CD31 immunofluorescence staining, using wound healing rate, granulation tissue growth, collagen volume ratio, epidermal thickness, and vascular and hair follicle regeneration as indicators.

All the animal experiments were approved by the institutional review board of Changchun University of Chinese Medicine.

# Supplementary Results

- 1. **Mechanical properties of hydrogel**

Cyclic compression experiments were conducted to characterize the elasticity of the hydrogels by evaluating their shape recovery. The experimental results showed that the shape recovery of PAAM/DA-SA/GMA-GEL hydrogels reached 92% at 37°C. In addition, the shape recovery of the hydrogels remained above 90% after five cycle tests, which showed good mechanical property.

**
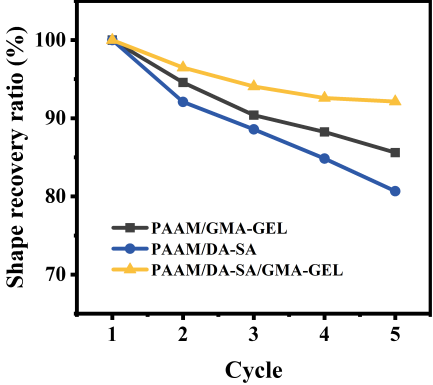
**

**Supplementary Fig. 1.** Shape recovery ratio of different hydrogels.

- 1. **Evaluation of hydrogel biocompatibility**

Cytotoxicity of the aforementioned hydrogels was determined using the leachate method. A significant increase in cell viability was observed within 3 days, suggesting robust cell growth during the experimental period (Fig. S1). On the day 3, the cell viability of PDGA hydrogel was significantly higher than that of PAAM/DA-SA/GMA-GEL and the control group (P<0.01).

**
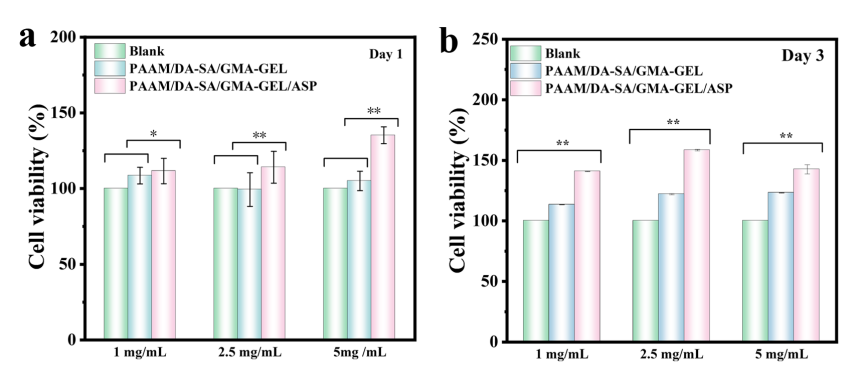
**

**Supplementary Fig. 2.** Cell compatibility assessment of the PDGA and PAAM/DA-SA/GMA-GEL hydrogel; (a) Cell viability on the first day of culture; (b) Cell viability on the third day of culture.
